# Machine-Learning Prediction of Extracellular Vesicle Protein Sorting Expands the Characterization of Secretory Functions in *Mucor circinelloides*

**DOI:** 10.3390/jof12060442

**Published:** 2026-06-17

**Authors:** João Neves-da-Rocha, Marcos E. R. Lopes, Lucas F. Nogueira, Shaghayegh Moghadam, Felipe E. A. De Paiva, Fausto Almeida

**Affiliations:** 1 Department of Biochemistry and Immunology, Ribeirão Preto Medical School, University of São Paulo, Ribeirão Preto 14049-900, SP, Brazil; lucasfarian@usp.br (L.F.N.); sgh.moghadam@usp.br (S.M.); felipeeduardopaiva@usp.br (F.E.A.D.P.); 2 Department of Genetics, Ribeirão Preto Medical School, University of São Paulo, Ribeirão Preto 14049-900, SP, Brazil; marcoserl@usp.br

**Keywords:** *Mucor circinelloides*, secretome, machine learning, extracellular vesicles, CAZymes, pathogenic fungi

## Abstract

Opportunistic infections are a growing concern in medical mycology. In that context, *Mucor circinelloides* is recognized as an etiological agent of emerging life-threatening infections in humans. Furthermore, this biomass-degrading species is important in the biotechnological context due to its potential for enzyme discovery. In this work, we describe an in silico pipeline for the comprehensive characterization of the secretome of this species. Our analyses suggest that *M. circinelloides* encodes a diverse enzymatic repertoire, including multiple carbohydrate-active enzymes (CAZymes). Functional characterization of this secreted protein set identified candidate proteins with potential relevance for industrial application and virulence-related processes. Importantly, we extended this framework by integrating a sequence-based machine-learning (ML) approach for the prediction of extracellular vesicle (EV)-associated proteins. Using a Random Forest model trained on high-confidence EV datasets, we predicted a subset of candidate proteins in *M. circinelloides* and explored their functional distribution. The predicted EV proteome displayed a degree of similarity to experimentally characterized human EV proteomes, suggesting that the model is able to capture sequence-derived predictive signals. Overall, our results support the potential of ML as a scalable strategy to be integrated into the study of fungal EVs. This approach provided a foundation for research aimed at experimentally validating EV cargo and refining predictive models. Beyond *M. circinelloides*, we provide a framework that may be useful for future studies on EV biology across diverse fungi of medical and biotechnological interest.

## 1. Introduction

Saprophytic fungal species play a key role in nutrient cycling and organic matter decomposition in terrestrial ecosystems, adding to the function of bacteria in forest and agricultural soils [1,2]. Among these fungi, species from the order Mucorales are recognized as important decomposers of complex organic substrates [3]. Mucorales are an early-diverging clade belonging to the phylum Mucoromycota and are phylogenetically distinct from both Ascomycota and Basidiomycota. Consequently, Mucorales are evolutionarily distant from Dikarya species, forming a basal lineage that retains several ancestral features such as coenocytic hyphae, zygospores, and chitosan-rich cell walls [4,5,6,7,8]. From a metabolic point of view, these species also exhibit fast hyphal extension rates associated with strong reliance on rapidly absorbed nutrient sources and a simpler genome architecture/regulation [9,10,11,12,13]. *Mucor circinelloides* is particularly noteworthy for its dual relevance as an emerging opportunistic pathogen and a cellular platform for biotechnological applications [14,15].

As the etiological agent of mucormycosis, *M. circinelloides* is associated with significant clinical risk, especially to immunocompromised patients, with mortality rates reaching 67% in individuals with decompensated diabetes mellitus [16,17,18,19]. As a particular clinical aspect of opportunistic mucormycotic infections, acquired resistance has been reported as an adaptive trend associated with epigenetic mechanisms, which include siRNA-dependent epimutations that confer resistance to antifungal treatments in vivo [20,21]. Virulence attributes are directly linked to dimorphic transitions in this species [22,23], and comparative genomic studies contrasting virulent to avirulent isolates revealed 773 genes that are either truncated, discontiguous, or completely absent in the NRRL3631 avirulent strain [9]. Genes potentially associated with virulence traits in *M. circinelloides* were enriched in functions such as protein maturation, post-translational modifications (PTMs), transport, vesicle formation, secretory pathway sorting, and also included many enzymes involved in the biogenesis of extracellular structures [24]. These observations indicate that surface and secreted functions can be particularly relevant to pathogenicity and host interaction in mucormycoses.

In eukaryotes, a major secretion mechanism canonically synthesizes proteins in the endoplasmic reticulum (ER) and further directs them for post-translational processing in the Golgi apparatus. Protein sorting into the ER occurs co-translationally and depends on the presence of a signal peptide in the N-terminal region of the translating protein [25,26]. Such signal peptides are known as Sec/SPI, referring to proteins that enter the classical secretory pathway and are cleaved by Signal Peptidase I after translocation into the ER [27,28]. Proteins undergo chaperone-assisted folding and initial modifications in the ER, while final maturation, including carbohydrate modifications, occurs in the Golgi. After maturation, Sec/SPI proteins are incorporated into the cell membrane or secreted to the extracellular medium by exocytosis [29,30,31,32,33,34]. While the ER-Golgi secretory pathway remains a leading export system in eukaryotic cells, organisms also rely on noncanonical strategies to deliver extracellular biomolecules.

Extracellular vesicles (EVs), in particular, emerge as important secreted structures that carry not only proteins as their cargo, but are also filled with lipids, polysaccharides, and RNAs [35,36]. The biogenesis of EVs has been reported to occur either through ESCRT or ceramide-dependent formation of intraluminal vesicles within multivesicular bodies [37,38,39,40,41]. Protein sorting into EVs, however, remains an incompletely understood process that is thought to occur through ubiquitination, lipid microdomain association, and specific PTMs, rather than canonical signal peptides [38,41,42,43,44,45,46]. The lack of conserved sequence-encoded determinants for EV protein sorting makes it more complex to determine whether putative extracellular proteins lacking classical Sec/SPI signals might be carried and delivered through these structures [47]. Therefore, studies focused on determining EV cargo almost exclusively rely on direct and sensitive experimental methods for biomolecule identification, such as mass spectrometry-based proteomics, RNA sequencing, and lipidomic analyses of purified vesicles. Limitations to these techniques include the requirement for highly purified vesicle preparations, the typically low amount of material recovered from EV isolations, the potential co-isolation of endogenous contaminants, and the fact that experimental detection is often condition-dependent, which may prevent the comprehensive identification of EV-associated molecules across different biological contexts [48,49,50,51,52,53]. Yet, a detailed characterization of EV-associated proteins is critical for a mechanistic interpretation of the functions and molecular organization of these secreted structures.

In fungi, EVs have emerged as critical elements for understanding key aspects of their biology. By transporting diverse cargo, these structures participate in processes such as stress response, intercellular communication, epithelial adhesion, and host–pathogen interactions [50,54,55,56,57,58,59,60]. EVs also play a direct role in modulating the host immune response, and advances in understanding EV structure and cargo have driven the development of vaccines, antifungal therapies, and diagnostic biomarkers [61,62,63,64]. Nevertheless, proteomic characterization of EV cargo remains challenging and demanding, which limits our comprehension of EV function in high-priority processes such as cell communication and infection. Therefore, computational prediction approaches constitute a valuable strategy for the large-scale characterization of EV-associated proteins in fungal genomes. Ongoing advances in artificial intelligence (AI) and machine learning (ML) have contributed to the in silico characterization of EV cargo and biomarkers [65,66,67,68,69,70]. Using established databases on proteins isolated from human EVs, a seminal study has demonstrated that computational prediction of EV-associated proteins is possible through sequence-based strategies [47]. Redirecting this approach to fungal species might contribute to our fundamental understanding of the secretome repertoire of diverse fungi, filling an important gap in the molecular and medical mycology literature.

In this work, we performed a comprehensive characterization of the secretome of *M. circinelloides*. This characterization was achieved using a sequential pipeline integrating multiple tools for the detection of Sec/SPI signal peptides, protein subcellular localization, and transmembrane topology. A rich secretory repertoire of carbohydrate-active enzymes (CAZymes) was identified in the dataset and provided meaningful insights into the ability of this species to degrade plant biomass and remodel cell structure during infection. In parallel, candidate EV-associated proteins were identified by a multicriteria Random Forest ML strategy that combined 76 sequence-based protein features (physicochemical, structural, and PTM features) associated with vesicle sorting. Predicted EV proteins displayed a functional profile consistent with experimentally validated human EV proteins. Together, these analyses provided a global view of the secretome of *M. circinelloides* and established a cutting-edge strategy for the prediction and large-scale characterization of EV cargo proteins in fungi.

## 2. Materials and Methods

### 2.1. Identification of Sec-Dependent Secreted Proteins

The complete proteome of *M. circinelloides* strain 1006PhL was retrieved from the Ensembl Fungi database [71] and screened for the occurrence of N-terminal signal peptides using SignalP v6.0 [28], employing the prediction model for eukaryotic organisms. All proteins containing a classical cleavable signal peptide consisting of a defined sequence that directs proteins to the ER via the Sec translocation pathway were retained. These Sec/SPI-positive proteins were then analyzed using the software DeepTMHMM v1.0 with default settings [72] to determine the number and position of transmembrane helices (TMHs). In the resulting dataset, all proteins containing TMHs were removed from the secretome dataset since they represent integral membrane proteins. Proteins with a single predicted TMH were maintained as part of the secretome only when helix position and topology were restricted to the protein N-terminal region corresponding to the signal peptide.

### 2.2. Functional Annotation of Carbohydrate-Active Enzymes

CAZyme candidates were identified through complementary sequence similarity and profile-based approaches. First, Hidden Markov Model (HMM) searches were conducted using HMMer [73,74] against the dbCAN3 HMM [75], which contains curated domain profiles representative of CAZyme families. In parallel, sequence similarity searches were performed using DIAMOND [76,77] against the CAZy database (http://www.cazy.org (accessed on 24 March 2026)) to detect homologous proteins with previously annotated CAZyme functions. The results obtained from both approaches were integrated into a single dataset without excluding predictions supported by only one method. For each protein candidate, the type and number of supporting tools were explicitly recorded to indicate the level of annotation support. Proteins identified by both HMMer/dbCAN and DIAMOND/CAZy searches are considered high-confidence CAZyme predictions. Finally, the functional classification of the predicted CAZymes into their respective classes and families was assigned according to the annotations provided in the CAZy database.

### 2.3. Identification of Sec-Independent Secreted Proteins

Non-classical protein secretion was assessed through a sequential multi-tool pipeline. Initially, the proteome of *M. circinelloides* was analyzed with DeepLoc v2.1 using default settings [78], a deep learning-based predictor of subcellular localization, and proteins predicted as extracellular were selected as primary candidates. To ensure exclusion of proteins following the canonical ER-Golgi secretory route, the initial extracellular set was further filtered by removing all proteins predicted by SignalP v6.0 [28] to contain N-terminal signal peptides, thereby restricting the dataset to extracellular proteins lacking classical secretion signals. The resulting candidates were subsequently analyzed with DeepTMHMM v1.0 [72] to exclude proteins containing any TMH. To further validate Sec-independent secretion, the filtered candidates were analyzed with SecretomeP v2.0 using default settings [79], an ML model for ab initio predictions of non-classical protein secretion, using a threshold of neural network (NN) score ≥ 0.5. Proteins flagged by SecretomeP as containing signal peptides were excluded regardless of their NN score. Proteins consistently supported by DeepLoc, DeepTMHMM, and SecretomeP were considered high-confidence candidates for non-classical secretion, while partially supported candidates were retained for exploratory analyses.

### 2.4. Machine-Learning Prediction of EV Cargo Proteins

ML-based prediction of EV protein cargo was conducted following a previously established workflow using sequence-based protein features [47]. In this approach, a supervised classification model was trained on a curated human protein dataset containing 5965 and 10,290 EV- and non-EV proteins, respectively. The underlying rationale of this methodology is that EV protein sorting is influenced by intrinsic physicochemical and structural properties encoded at the protein sequence level, which can be learned by AI models and generalized across eukaryotic systems. Feature generation followed a comprehensive and standardized bioinformatics pipeline integrating multiple layers of sequence-derived information. Primary sequence features included amino acid composition normalized by protein length, global physicochemical descriptors such as molecular weight, aromaticity, instability index, grand average of hydropathy (GRAVY), isoelectric point, and net charge at different pH values. Structural and biophysical features were incorporated from predictions generated by NetSurfP v3.0 [80], including secondary structure elements, relative surface accessibility, and disorder propensity, which collectively capture folding state and surface exposure patterns relevant to protein sorting. Membrane association and topology were inferred using DeepTMHMM v1.0 [72], allowing for discrimination between soluble and membrane-embedded proteins. PTM features were derived from predictions generated by MusiteDeep [81], a deep learning-based model, and included major PTM classes such as phosphorylation, glycosylation, ubiquitination, acetylation, methylation, and lipid-related modifications. All protein features were generated using the same scripts, tools, and settings from the original training dataset [47]. The resulting test dataset, containing 76 features, was systematically normalized and transformed to ensure compatibility with the original training distribution, preserving the statistical properties required for model generalization. The ML model consisted of a Random Forest classifier implemented in scikit-learn, which leverages an ensemble of decision trees to capture non-linear relationships between features [47]. This algorithm provides robustness to feature heterogeneity, resistance to overfitting, and the ability to model complex interactions. The trained model was subsequently applied to the complete proteome of *M. circinelloides* to generate probabilistic predictions of EV cargo, enabling large-scale prediction of potential EV-associated proteins.

### 2.5. Orthology and Functional Validation of Machine-Learning Predictions

To evaluate ML predictions, we performed a functional analysis of the top 5% highest-scoring EV-associated candidate proteins in *M. circinelloides* (*n* = 663), which were annotated and classified into biological categories based on homology searches [82] and their subcellular localization [78]. The resulting functional and localization profiles were compared to those obtained from a curated dataset of 2649 high-confidence human EV proteins previously compiled from studies employing high-purity EV isolation methods and mass spectrometry [47]. Comparative analyses allowed us to assess whether sequence-based ML predictions recapitulated major biological features from experimentally validated EV proteomes. As an independent quantitative approach, an orthology-based analysis was performed using experimentally characterized EV proteins from the ascomycete species *Histoplasma capsulatum* [83]. Initially, the 5% highest-scoring proteins predicted as EV-associated in *M. circinelloides* were compared against a dataset of 1110 proteins previously identified in EVs of *H. capsulatum* by proteomic analyses (EVpedia accession number 273037290101). Orthologous relationships were inferred using OrthoFinder v3.1.2 [84]. The number of proteome-wide and EV-associated proteins in *M. circinelloides* exhibiting orthologous relationships with the *H. capsulatum* EV dataset was quantified to assess the expected orthology occurrence in a sample size of *n* = 663 under a null model. Empirical statistical significance was assessed through a permutation test in which 100,000 random samples of 663 proteins were drawn from the complete *M. circinelloides* proteome. The resulting null distribution was combined with hypergeometric testing to evaluate significance, providing a statistical validation of the predictive capacity of the ML model.

## 3. Results

### 3.1. Global Characterization of the Sec-Dependent Secretome of Mucor circinelloides

**Figure 1:**
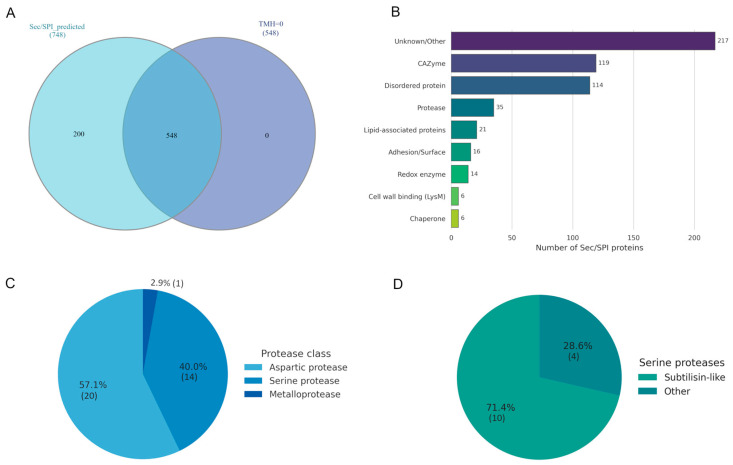
Characterization of the classical secretome of *Mucor circinelloides*. (**A**) Venn diagram showing the overlap between Sec/SPI-positive proteins predicted by SignalP v6.0 and proteins lacking transmembrane helices (TMH = 0) as identified by DeepTMHMM, defining the core classical secretome. (**B**) Horizontal bar plot summarizing the main functional categories of Sec/SPI-positive proteins and their respective abundances based on annotation. (**C**) Pie chart depicting the distribution of protease classes within the secretome. (**D**) Pie chart showing the subclass distribution of serine proteases, indicating the predominance of subtilisin-like enzymes.

To characterize the repertoire of secreted proteins of *M. circinelloides*, we initially focused on the identification of proteins that enter the classical ER-Golgi secretory pathway. The complete proteome of *M. circinelloides* strain 1006PhL was screened for the presence of N-terminal Sec/SPI signal peptides using the deep learning-based tool SignalP v6.0. This analysis indicated a total of 748 Sec/SPI-positive proteins, which were subsequently filtered using DeepTMHMM to exclude proteins containing transmembrane helices. A subset of 548 secreted proteins was identified as the core classical secretome, while 200 proteins were classified as integral membrane components (Figure 1A). Stepwise results and the complete list of proteins identified in this analysis are provided in Supplementary Material Table S1.

Functional annotation of Sec/SPI-positive proteins suggested that the classical secretome may be enriched in carbohydrate-active functions and proteases. Beyond these major classes, the ER–Golgi secretome also included proteins associated with lipid metabolism, adhesion, redox processes, chaperone activity, and LysM domain-containing proteins (Figure 1B). Concerning the set of secreted proteases, this class was dominated by aspartic proteases, followed by serine proteases and a small subset of metalloproteases (Figure 1C). The subset of serine proteases was dominated by subtilisin-like enzymes (Figure 1D), which are critical in the context of animal host-tissue invasion.

Notably, a substantial fraction of the identified proteins lacked detectable homology to characterized entries in the InterPro database, which may reflect species-specific or uncharacterized functions. In parallel, multiple proteins were annotated only at the feature level and displayed intrinsically disordered signatures (Figure 1B). Proteins categorized as non-core functional classes were also mostly annotated at the feature level and thus correspond to partially characterized domains and families. Overall, the high proportion of hypothetical and incompletely characterized proteins in the secretome of *M. circinelloides* likely reflects its status as a non-model species while also supporting potentially novel functions and biotechnological applications.

### 3.2. Mucor circinelloides Secretes an Enzymatic Repertoire Enriched in CAZymes with Potential Biotechnological Applications

Given the high prevalence of CAZymes in the dataset of secreted proteins and their known relevance to processes related to host interaction and plant biomass degradation, we performed a comprehensive characterization of this class of enzymes. Validated annotations were achieved using homology-based HMMER searches against the dbCAN HMM database and DIAMOND searches against the CAZy sequence database. Results from both tools were integrated without excluding single-tool predictions, and the level of support for each protein was explicitly recorded (Supplementary Material Table S2). The resulting set of CAZymes was categorized into six major classes according to the classification scheme provided by the CAZy database.

**Figure 2:**
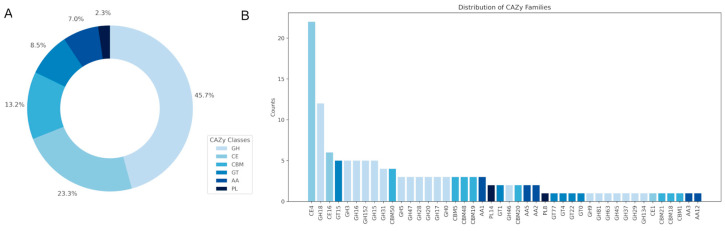
Classification of CAZymes in the secretome of *Mucor circinelloides*. (**A**) Donut chart illustrating the proportional distribution of CAZy classes based on the total number of identified CAZyme proteins. (**B**) Bar plot showing the abundance of individual CAZy families within the Sec/SPI-positive dataset.

A total of 119 Sec/SPI-positive CAZymes were identified in the genome of *M. circinelloides*. CAZy categorization included all six major enzyme classes. Glycoside Hydrolases (GH) formed the most abundant CAZy class, followed by Carbohydrate Esterases (CE), Carbohydrate-Binding Modules (CBM), Glycosyltransferases (GT), Auxiliary Activities (AA), and Polysaccharide Lyases (PL) (Figure 2A). GHs also formed the most diverse CAZy class in the number of representative families. Among these, GH18, GH3, GH16, GH152, and GH15 had a prominent representation in the dataset. Nonetheless, the single most abundant CAZy family among Sec/SPI-positive CAZymes was CE4 (Figure 2B). The distribution of CBMs was restricted to the GH18, GH15, and GH45 families, contributing to substrate ligation and specificity of these enzymes. CBMs were also found in CAZymes with unassigned classes (Supplementary Material Table S2).

Functional annotation of CAZy families suggested a marked predominance of enzymes involved in cell wall remodeling and plant polysaccharide degradation in *M. circinelloides*, followed by functions related to glycan biosynthesis, CBM-mediated substrate targeting, and auxiliary oxidative activities (Table 1). This functional distribution is consistent with a saprophytic lifestyle, supporting the ability to utilize complex plant-derived substrates while also reflecting cell wall dynamics that may be critical for pathogenic attributes of this species. As an overview, this analysis supported the identification of specific proteins with potential relevance in biotechnological and medical contexts (see Discussion). To further explore their structural properties, we performed AlphaFold-based predictions for representative CAZymes across all six major classes. The resulting models displayed highly confident and well-defined structures, as indicated by low Predicted Aligned Error (PAE) values and consistent folding patterns (Figure S1).

### 3.3. Characterization of Sec-Independent Extracellular Proteins

**Figure 3:**
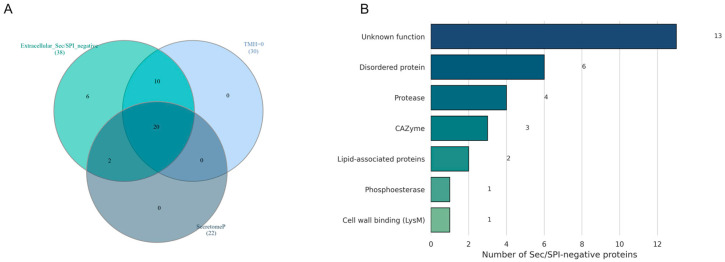
Characterization of Sec-independent extracellular proteins of *Mucor circinelloides*. (**A**) Venn diagram showing the overlap between Sec/SPI-negative proteins predicted by DeepLoc after SignalP filtering, proteins lacking transmembrane helices (TMH = 0) as identified by DeepTMHMM, and SecretomeP predictions of non-classical secreted proteins. (**B**) Horizontal bar plot summarizing the main functional categories of Sec/SPI-negative proteins and their respective abundances based on annotation.

We further evaluated protein subcellular localization to identify extracellular proteins that are secreted in a Sec-independent manner. This strategy consisted of a sequential multi-tool pipeline. An initial set of 38 extracellular Sec/SPI-negative proteins was identified using SignalP filtering and DeepLoc, a deep learning-based predictor of subcellular localization. This set corresponded to predicted extracellular proteins that lacked classical signal peptides, indicating their secretion through non-classical pathways. To exclude membrane-associated proteins, a second filter was applied using DeepTMHMM, resulting in a final set of 30 extracellular Sec/SPI-negative proteins (Figure 3A). The dataset was also analyzed using SecretomeP, which confirmed that 20 of these proteins had a moderate non-classical secretion signature (Table A1).

Functional annotation suggested that a large fraction of the dataset lacked characterized functional orthologs, including many SecretomeP high-scoring proteins. Additionally, several proteins containing disordered signatures were also reported. Functional categories included four proteases, three GH CAZymes (GH16, GH18, and GH46), two lipid-associated proteins, one phosphoesterase, and one LysM domain-containing protein (Figure 3B). The complete multi-tool sequential results produced in this analysis are available in Supplementary Material Table S3.

The six proteins predicted to contain disordered regions were structurally analyzed in IUPred3 and AlphaFold (Figure S2). Overall, these proteins exhibited extensive disorder, with large portions of their sequences yielding low-confidence structural models, as supported by low pLDDT scores and high PAE values, consistent with intrinsically disordered behavior. Notably, EPB81863 displayed a partially structured region containing well-defined α-helices and β-sheets, suggesting the presence of a potential functional core. EPB82347 showed a small region with secondary elements (α-helices and β-sheets), although with limited confidence. EPB82275 was predicted to be entirely intrinsically disordered, forming a large protein lacking stable tertiary structure. Similarly, EPB7963 and EPB92248 corresponded to smaller proteins that were almost entirely disordered. In contrast, EPB87092 produced a large and very complex structure, but with uniformly low confidence scores, indicating a flexible and unresolved conformation (Figure S2).

### 3.4. Machine-Learning Prediction of EV Cargo Proteins

**Figure 4:**
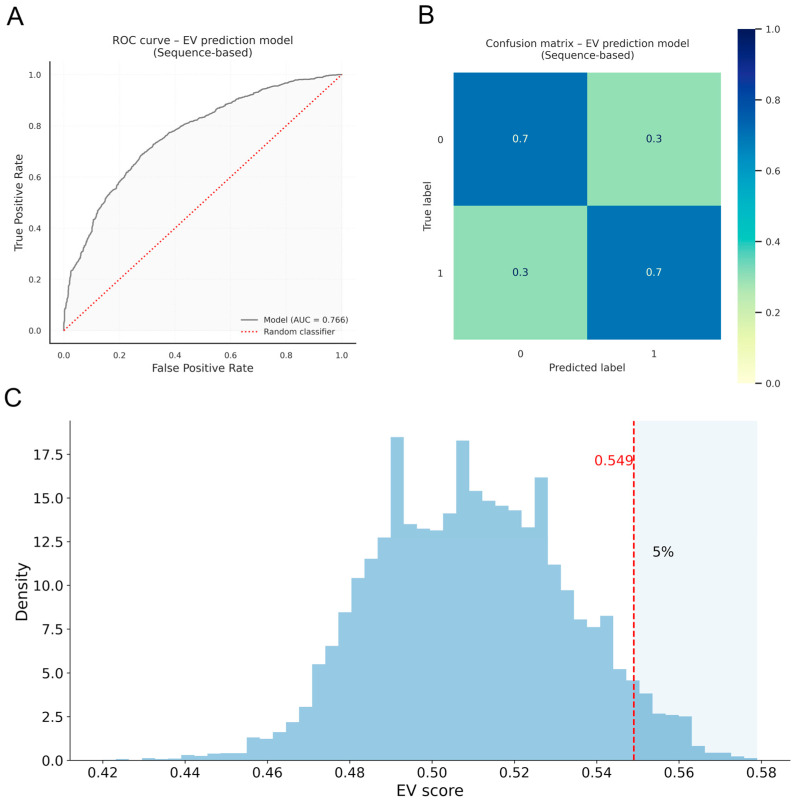
Sequence-based machine-learning prediction of extracellular vesicle (EV) proteins. (**A**) Receiver operating characteristic (ROC) curve showing the performance of the Random Forest model, with the corresponding area under the curve (AUC) indicating its discriminative power. The red dashed line represents a theoretical random classifier. (**B**) Normalized confusion matrix illustrating the classification performance of the model on the validation dataset. Model training and validation were performed on two independent curated datasets of human proteins, filtered to include true-positive proteins identified exclusively by mass spectrometry using high-purity EV isolation methods [47]. (**C**) Distribution of predicted EV scores across the proteome of *Mucor circinelloides*, indicating the cutoff corresponding to the top 5% highest-scoring proteins (red dashed line), which were selected as candidate EV-associated proteins for exploratory analyses.

To explore the feasibility of predicting EV-associated proteins in fungi using sequence-based features, we applied a previously established ML model [47] to the proteome of *M. circinelloides*. This analysis was designed as an exploratory approach to evaluate whether a methodology originally trained on human data could capture biologically meaningful signals in fungi, thereby addressing a major unresolved challenge in the field: the lack of reliable predictors for EV-associated proteins. Random Forest model performance, as assessed on the original human validation dataset, showed moderate discriminative performance. The receiver operating characteristic (ROC) curve (Figure 4A) yielded an area under the curve (AUC) of 0.766, indicating that the model can effectively distinguish EV-associated from non-EV proteins. Consistently, the normalized confusion matrix (Figure 4B) suggested a satisfactory classification performance, with true positive and true negative rates of 0.7, and corresponding false positive and false negative rates of 0.3. This balanced behavior was further supported by class-wise metrics, with precision values of 0.700 and 0.702, recall values of 0.704 and 0.698, and F1-scores of 0.702 and 0.700 for non-EV (class 0) and EV-associated proteins (class 1), respectively. These results suggest that the model captures generalizable sequence-level features associated with EV targeting.

We next applied the Random Forest model to the proteome of *M. circinelloides* to obtain EV prediction scores (Figure 4C). Across the analyzed proteome (*n* = 12,227 proteins), EV scores ranged from 0.42 to 0.579, with a mean of 0.51 and a standard deviation of 0.023. The interquartile range was similarly restricted (Q1 = 0.492; median = 0.509; Q3 = 0.526), indicating a high concentration of predictions within a limited score interval. Based on this distribution, a percentile-based thresholding strategy was adopted to prioritize high-confidence EV candidates. We highlighted the top 5% of proteins according to their EV scores for the identification of the most probable EV-associated proteins while avoiding arbitrary cutoffs near the central tendency of the distribution. We decided to adopt a conservative approach for candidate selection since the distinction between EV and non-EV proteins was gradual rather than discrete, based on the features analyzed. The complete test dataset consisting of 76 protein features assigned to the proteome of *M. circinelloides* is available in Supplementary Material Table S4.

### 3.5. Comparative Genomics and Functional Annotation Support Machine-Learning Predicted EV Cargo

To gain insight into the biological composition of the predicted EV-associated proteome, we functionally annotated the top 5% highest-scoring candidates in *M. circinelloides* (*n* = 663) and compared their profiles with a curated dataset of 2649 high-confidence human EV proteins from the original training dataset [47] (Supplementary Material Table S5). This comparative framework was used to assess whether sequence-based ML predictions recapitulate known features of experimentally validated EV proteomes.

**Figure 5:**
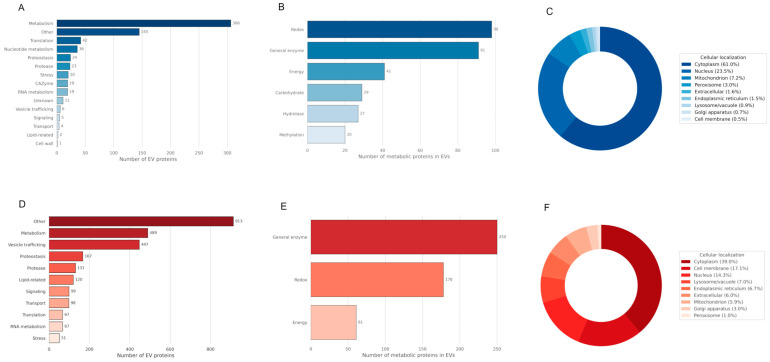
Functional and subcellular characterization of predicted and experimentally supported extracellular vesicle (EV)-associated proteins. Panels (**A**–**C**) represent the top 5% highest-scoring EV candidates identified in the proteome of *Mucor circinelloides* based on machine-learning (ML) predictions. (**A**) Distribution of proteins across major functional categories. (**B**) Composition of metabolic subcategories within the fungal EV dataset. (**C**) Proportional distribution of subcellular localizations predicted using DeepLoc v2.1. Panels (**D**–**F**) depict the corresponding analyses for a curated dataset of 2649 high-confidence human EV proteins, defined by identification through mass spectrometry and high-purity EV isolation methods. (**D**) Functional classification across major categories. (**E**) Distribution of metabolic subcategories. (**F**) Subcellular localization profile based on a representative subset of 500 proteins used for sampling. These analyses supported a qualitative comparison of functional patterns between ML-predicted fungal EV candidates and experimentally supported human EV proteins.

Functional classification of the fungal EV candidates suggested a partial predominance of proteins involved in central metabolic and biosynthetic processes (*n* = 306), followed by proteins of varied or general function (other, *n* = 145), along with categories such as translation (*n* = 42), nucleotide metabolism (*n* = 36), proteostasis (*n* = 24), and proteases (*n* = 23). Additional categories included stress response, CAZymes, RNA metabolism, vesicle trafficking, signaling kinases, transport, lipid-related, and cell wall (Figure 5A). Interestingly, EV protein candidates included a reduced number of uncharacterized and intrinsically disordered proteins (Supplementary Material Table S4). An in-depth analysis of the dominating metabolic category further indicated a composition enriched in redox-related enzymes (*n* = 98) and general metabolic enzymes (*n* = 91), followed by proteins involved in energy metabolism (*n* = 41), carbohydrate metabolism (*n* = 29), hydrolase activity (*n* = 27), and methylation processes (*n* = 20) (Figure 5B). Subcellular localization predictions indicated a dominant cytoplasmic origin for the fungal EV candidates (61.0%), followed by nuclear-associated proteins (23.5%) and a smaller fraction of mitochondrial proteins (7.2%). Components of the classical secretory pathway, such as endoplasmic reticulum-associated proteins, were comparatively underrepresented (Figure 5C).

To evaluate the congruence between experimentally validated and ML-predicted EV profiles, we compared the functional distribution of fungal proteins to the dataset of human EV proteins characterized by mass spectrometry in studies using high-purity EV isolation methods [47]. As reported for *M. circinelloides*, human EV proteins also showed a substantial contribution from metabolic functions (*n* = 489), although the largest category corresponded to proteins grouped as ‘other’ (*n* = 913), representing a heterogeneous set of proteins associated with either general or highly specialized cellular functions that could not be assigned to major classes. This broader distribution likely reflects both the higher functional diversification of the human proteome and the greater depth of functional annotation available for mammalian proteins (Figure 5D). Vesicle trafficking proteins (*n* = 447) were more abundant in the human dataset than in fungal predictions, supporting a stronger representation and possibly the better annotation status of EV components in the human proteome. Still, the overall landscape from predicted fungal EV proteins partially recapitulated the functional distribution of experimentally validated EV cargo (Figure 5A,D). Within metabolic subcategories, human EVs similarly displayed a dominance of general enzymes (*n* = 250) and redox-related proteins (*n* = 178), with energy metabolism contributing to a lesser extent (*n* = 61) (Figure 5E). Subcellular localization patterns also showed a consistent overlap between the two datasets, with cytoplasmic proteins contributing to the larger fraction of EV cargo (Figure 5F). Notably, membrane-associated and endomembrane system proteins were more prominent in human EVs. The consistency of the results obtained supports the potential of continued ML advances for the characterization of complex biological processes in fungi, particularly protein sorting into EVs.

**Figure 6:**
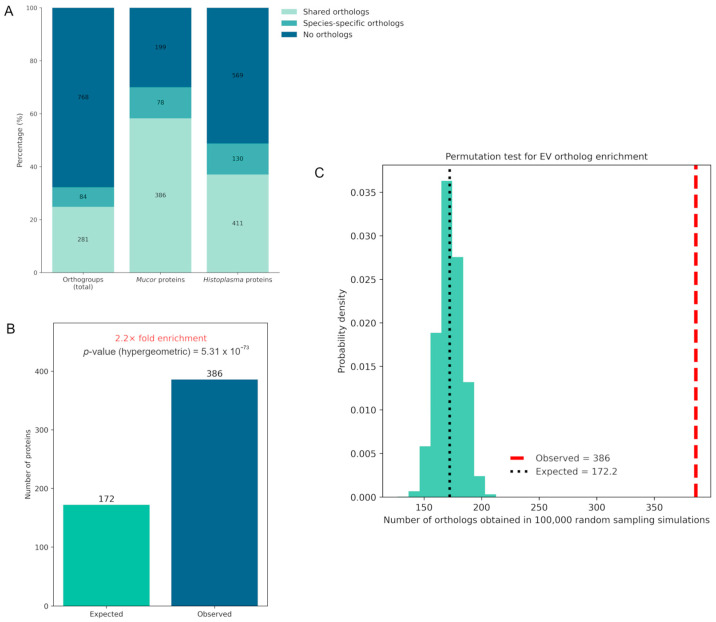
Orthology-based validation of machine-learning (ML) predictions contrasting the 5% highest-scoring extracellular vesicle (EV)-associated candidates in *Mucor circinelloides* (N = 663 proteins) against a dataset of 1110 proteins experimentally identified in EVs from *Histoplasma capsulatum*. (**A**) Distribution of orthology categories identified by OrthoFinder. Stacked bar plots show the proportions of orthologues that are shared between *M. circinelloides* and *H. capsulatum*, species-specific orthologues, and proteins without orthologues within the two datasets. Values inside each bar indicate the number of orthogroups or proteins assigned to each category. (**B**) Enrichment analysis comparing the observed number of *M. circinelloides* proteins with orthologues among EV-associated proteins of *H. capsulatum* against the number expected under a random model. (**C**) Null distribution of shared orthologues generated by permutation analysis. A total of 100,000 random samplings of 663 proteins were performed from the complete *M. circinelloides* proteome (12,611 proteins), and the number of proteins expected to possess orthologues within the EV-associated dataset of *H. capsulatum* (1110 proteins) was recorded for each iteration. The red dashed line indicates the observed number of orthologues (386), whereas the black dotted line indicates the mean value expected under the null model (172.2). The observed enrichment strongly supports the ML model predictive capacity in fungal datasets.

As an independent validation strategy combining evolutionary aspects to statistical discrimination, we next investigated whether the EV-associated proteins predicted in *M. circinelloides* recapitulated experimentally characterized orthologues from the EV proteome of another fungal species. To this end, the top 5% highest-scoring EV candidates (663 proteins) were compared against a dataset of 1110 proteins identified through mass spectrometry in *H. capsulatum* EVs. Orthology inference revealed a substantial degree of conservation between the two EV datasets (Figure 6A, Supplementary Material Table S6). Among the 1133 orthogroups identified, 281 (24.8%) contained proteins from both species and were classified as shared orthogroups, whereas 84 (7.4%) corresponded to species-specific multigene orthogroups and 768 (67.8%) were represented by singletons lacking detectable orthologues within the datasets. At the protein level, 386 of the 663 predicted EV-associated proteins from *M. circinelloides* (58.2%) were assigned to orthogroups shared with *H. capsulatum*, while 78 proteins (11.8%) belonged to species-specific multigene families and 199 proteins (30.0%) lacked detectable orthologues. Similarly, among the experimentally identified EV proteins from *H. capsulatum*, 411 proteins (37.0%) were associated with shared orthogroups, 130 (11.7%) belonged to species-specific multigene families, and 569 (51.3%) were classified as proteins without detectable orthologues. These results indicate that a substantial fraction of the predicted *M. circinelloides* EV proteome belongs to evolutionarily conserved protein families found in experimentally characterized fungal EV cargo.

We performed a permutation-based enrichment analysis to determine the statistical significance associated with ML model reliability based on the observed occurrence of orthologues. Considering the proteome size of 12,611 proteins for *M. circinelloides* and the 1110 proteins identified in *H. capsulatum* EVs as the reference set, 100,000 random samples of 663 proteins were generated to estimate the null expectation of orthologue occurrence. Estimates were performed based on a total of 3275 proteins in *M. circinolloides* that share homology with the reference *H. capsulatum* set (Supplementary Material Table S6). Under this scenario, and assuming a random classifier, only 172.2 proteins were expected on average to exhibit orthology relationships with the *H. capsulatum* EV dataset (Figure 6B,C). In contrast, the ML predictions yielded 386 orthologous proteins, corresponding to a 2.24-fold enrichment over the random expectation. The null distribution obtained from the permutation analysis was narrowly centered around the expected value, whereas the observed occurrence was located far outside the distribution range, producing an empirical random probability effectively indistinguishable from zero (Figure 6B,C). Consistently, hypergeometric testing indicated that such an overlap is extremely unlikely to occur by chance (*p* = 5.31 × 10^−73^), supporting the biological relevance of the predicted EV proteome. These results strongly reject the null hypothesis of a random classifier, demonstrating that the ML model effectively captured sequence-derived features that are truly associated with EV protein sorting in fungi.

## 4. Discussion

The global emergence of opportunistic pathogens is indicated by the first WHO report dedicated to tests and treatments for fungal diseases, which emphasizes the lack of new antifungals, limitations in diagnosis, and the emergence of resistant strains [85]. In the context of fungal–host interactions, pathogenic species rely on a repertoire of hydrolytic enzymes to invade host tissues [86,87,88]. In addition, secreted proteins participate in structural processes such as cell wall remodeling and extracellular matrix plasticity, which are critical for host interaction and resistance traits [89]. In biotechnology, the characterization of microorganism-derived enzymes is a fundamental step for biomass conversion and optimizing industrial processes [90,91]. At the same time, addressing virulence strategies contributes to our fundamental understanding of pathogens, aiding the development of novel therapeutic approaches against fungal infections [92].

Mucoromycota fungi have adapted to thrive in different environments and developed strategies to infect a wide variety of hosts, ranging from plants to animals [3,8]. *M. circinelloides* is one of the most common species of the order Mucorales and is also the etiological agent of life-threatening infections to humans. Studies have demonstrated that virulence attributes in this species are associated traits such as rapid growth, spore size dimorphism, and flexible carbon metabolism [9,93,94]. In that context, secreted proteins are particularly important for tissue invasion and overcoming host defenses [95,96,97]. Functional genomic insights into secreted proteins thus provide a global and straightforward view into infection mechanisms and unveil the biotechnological potential of overlooked fungal species. In this work, we implemented a comprehensive workflow for the identification and characterization of the complete set of secreted proteins of the opportunistic pathogen *M. circinelloides*. Additionally, prediction of EV-associated proteins was achieved using an ML Random Forest model based on protein physicochemical, structural, and PTM features.

Secretory processes rely on dedicated pathways that couple protein synthesis and modification to their extracellular delivery, thereby supporting functions such as nutrient acquisition, tissue adhesion, and biofilm formation [25,29]. Our results suggested that both the classical and non-classical secretomes of *M. circinelloides* are dominated by carbohydrate-active and proteolytic functions. The set of secreted proteases was dominated by aspartic proteases, followed by serine proteases and a smaller subset of metalloproteases. Aspartic proteases are typically active under acidic conditions and are often implicated in extracellular protein digestion, nutrient acquisition, and the processing of host-derived substrates [98,99]. Serine proteases, in turn, comprise a diverse group of enzymes involved in protein turnover, signaling, and interactions with the host environment [100,101]. Notably, this subset was largely dominated by subtilisin-like enzymes, which are widely recognized for their ability to degrade structural components of animal tissues, such as the extracellular matrix, thereby playing a key role in pathogenicity [12,102]. In contrast, metalloproteases, although less abundant in the results, are known to participate in the degradation of complex protein substrates and contribute to host invasion and virulence through metal ion-dependent catalytic mechanisms [103,104,105].

CAZymes comprised the most abundant class of secreted proteins in *M. circinelloides*. Several enzyme families, including GH18 (chitinases), GH20 (*β*-N-acetylhexosaminidases), CE4 (chitin deacetylases), and GH72 (*β*-1,3-glucanosyltransferases), are involved in fungal cell wall remodeling, contributing to structural maintenance as well as to pathogenic transitions [106,107,108]. In addition, the presence of GH28, which catalyzes the hydrolysis of pectin, a major acidic heteropolysaccharide in the plant primary cell wall and middle lamella, and GH45, involved in cellulose and hemicellulose degradation, supports a saprophytic lifestyle and the ability to exploit complex plant-derived substrates [106,109]. Consistently, plant polysaccharide-degrading activities were also identified in CE16 proteins, primarily known as hemicellulose acetyl esterases. These enzymes are often found in filamentous fungi and are notable for their ability to act on complex, acetylated hemicelluloses, including xylan, galactoglucomannan, and xyloglucan [106,110]. Beyond degradation, several CAZyme families, including GH15, GH31, and GH47, together with glycosyltransferases such as GT1 and GT15, are implicated in glycan metabolism [106,111]. The secretome also harbored AA enzymes, including AA1, AA2, AA3, AA5, and AA12, which are commonly associated with oxidative processes, stress responses, and secondary metabolism [112,113]. Ultimately, the presence of multiple CBMs, such as CBM5, CBM19, CBM20, CBM48, and CBM50, underscores the importance of substrate recognition to enhance enzymatic efficiency within the secretome of this species [114,115].

In fungi, the repertoire of secreted CAZymes determines adaptative features, enabling the degradation of plant biomass and competition for nutrients [116]. Mucoromycota fungi, such as *Rhizopus* spp., exhibit a distinctive enzymatic profile, with an abundance of the GH18 and CE4 families, involved in fungal cell wall remodeling [11,117]. In *M. circinelloides*, the expression of carbohydrate transporters and cell wall enzymes is influenced by the environmental conditions [118]. In *Mucor lusitanicus*, the ability to switch between filamentous (aerobic) and yeast-like (anaerobic) growth is also associated with significant changes in the expression of genes encoding CAZymes [119]. During the transition to anaerobiosis, 12 CAZyme genes are upregulated, including chitin deacetylases (CE4) and chitinases (GH18), while 20 are downregulated, including chitin synthases [119]. These expression changes suggest that the cell wall undergoes active remodeling during morphological transitions, with increased deacetylation of chitin to form chitosan, a more flexible polymer associated with the yeast-like form.

An additional aspect of the secretome of *M. circinelloides* was the presence of multiple proteins lacking functional annotation or matching orthologs of known function. This aspect indicates the potential of basal fungal species in harboring proteins that are currently uncharacterized, which might play important functions in the medical or biotechnological contexts. Notably, a large fraction of these proteins exhibited intrinsically disordered sequence signatures. Indeed, studying the function of intrinsically disordered proteins can be a challenging task and requires deeper insights about their conformational dynamics. According to the DisProt database, intrinsically disordered proteins perform a wide range of biological functions that arise either from their flexible, unstructured state or from disorder-to-order transitions upon binding [120,121]. These proteins are frequently involved in protein–protein and protein–DNA interactions, acting as hubs in molecular recognition and signaling. Their structural plasticity also supports roles as flexible linkers or entropic spacers, facilitating domain movement and macromolecular assembly. Additionally, disordered proteins often contribute to regulatory processes such as phosphorylation and acetylation. Other functions include chaperone-like activity, metal ion binding and detoxification, polymerization, and substrate recognition [120,121]. Continued efforts are required to unveil the range of functionality of these proteins in the secretome of *M. circinelloides*.

Research into fungal EVs has proven to be a cutting-edge strategy for investigating mechanisms of cell communication, environmental adaptation, and virulence in these organisms. EVs are known to act as transport vehicles for biomolecules, including proteins, lipids, polysaccharides, and RNAs, capable of modulating the fungal microenvironment [50,54,64]. In pathogenic fungi, EVs have been implicated in processes such as cell wall remodeling, biofilm formation, and the transport of metabolites and hydrolytic enzymes [62,122,123,124]. Importantly, EVs also emerge as key components in modulating the host immune response [125]. Since protein sorting into EVs does not rely on explicit sequence-encoded signals, as observed for signal peptides that target proteins to the canonical ER-Golgi pathway, bioinformatic predictions of EV cargo remained a long-persisting challenge [47]. Recent advances in ML- and AI-based strategies have driven outbreaks in the identification of complex, multifactorial, or indirect biological features that contribute to various cellular processes [126,127,128,129].

Based on Random Forest ML models, a pioneering study demonstrated that EV association is indeed feasible using deep learning strategies [47]. This study was centered on comparative models constructed based on different training datasets. The first dataset included human EV proteins collected from databanks without applying any filter, while the second dataset included a filter for EV proteins detected through mass spectrometry, and the third one included a filter for proteins detected exclusively through mass spectrometry using high-purity EV isolation methods. The third ML training dataset was pointed out by the authors as the most suitable for predicting EV-associated proteins that are most likely to be detectable by mass spectrometry [47]. Validation of this dataset further contrasted different ML scenarios: one first model trained using sequence-based and annotation-based PTM features, and one second model trained exclusively on sequence-based protein features. A direct comparison of the two models revealed that the inclusion of experimentally annotated features recovered from PTM/Processing information available in UniProt substantially contributed to model performance metrics [47]. Therefore, the sequence-based-only ML model partly lost precision–recall capacity.

Applying annotation-based PTM features for the construction of fungal datasets would, however, not be feasible since virtually all fungal species lack proteome-wide experimental data on PTMs. Therefore, we advocate that the prediction of EV proteins in fungi should rely on continued efforts to increase the sensitivity of sequence-based ML models. In particular, the analysis of feature importance in the two models revealed that the annotated palmytoilation PTM was the single most relevant feature to determine EV association. In addition, MusiteDeep predictions for S-palmytoilation showed a notable amount of false-positive and false-negative hits, failing to recover biological patterns based on experimentally annotated PTMs. At the same time, we noted that alternative lipidation-like predictors such as Palm-Pred and GPS-Palm displayed PTM predictions that could potentially perform better and were not included in this model [47]. To consider other possibilities for the continued evolution of sequence-based models, future studies should contrast the performance of qualitative and quantitative approaches to PTM sites, while also searching to include novel protein features potentially associated with EV sorting. Most importantly, we acknowledge that a critical step to improve EV predictions in fungi is to construct robust training and validation ML datasets based on experimentally validated fungal EV proteins. This approach could potentially capture the nuances of fungal-specific EV patterns with greater success, contributing to its implementation across species.

The application of a sequence-based ML model to the proteome of *M. circinelloides*, as performed in this study, represents a pioneering systematic attempt to predict EV-associated proteins in fungi. For that reason, this analysis was designed with an exploratory scope, aiming primarily to determine whether ML models could capture biologically meaningful patterns when transferred across eukaryotic systems. A major observation derived from this approach was a high degree of similarity between the predicted *M. circinelloides* EV proteome and experimentally validated human EV datasets. Despite differences in organismal complexity and annotation depth, both systems displayed broadly comparable functional trends, as well as consistent subcellular localization profiles. These observations showed that sequence-derived features governing EV cargo composition can be captured through ML models applied to fungal proteomes.

Indeed, orthology analysis based on experimentally validated fungal EV proteins provided robust support for ML model predictive capacity. Despite the evolutionary distance between Mucoromycota and Ascomycota, 386 predicted *M. circinelloides* proteins (58.2% of all candidates) belonged to orthogroups shared with experimentally detected EV proteins from *H. capsulatum*. Such observation substantially strengthened ML model reliability: the 2.24-fold increase over random expectation of orthologue occurrence (*p* = 5.31 × 10^−73^) demonstrated that the predicted protein set was highly deterministic and strongly directed toward evolutionarily conserved EV features. Importantly, because this validation was performed using an independent dataset from a phylogenetically distant fungal species, the observed enrichment supports the conservation of molecular determinants underlying EV cargo composition in fungi. These findings indicate that ML approaches are thus capable of capturing biologically meaningful determinants of EV association, highlighting their potential for EV characterization across fungi. From such a paradigm, it becomes possible to discuss and propose solutions for advancing the study of EVs, including strategies for delimiting optimized protein features and fungal-specific training datasets for ML-based predictions, which might significantly improve performance metrics such as accuracy in true-positive and true-negative discovery rates.

The distribution of prediction scores across EV candidate proteins suggested a relatively narrow and continuous range, indicating that the model did not sharply separate EV from non-EV proteins but instead assigned probabilistic scores within a constrained interval. This pattern was consistent with a conservative classifier operating on distributed sequence signals, where EV association is not defined by discrete attributes but rather by combinatorial features. In that context, the model may be more appropriately interpreted as a ranking tool for prioritizing candidate proteins than as a strict binary classifier. Importantly, the ability of the model to recapitulate major orthology, functional, and cell localization patterns from experimentally characterized EV proteomes reinforces the potential of sequence-based ML approaches as a viable strategy. In this sense, the present study can be viewed as a proof-of-concept that provides an initial indication of the feasibility of this methodological approach. The results obtained establish a foundation for future efforts aimed at refining predictive models, incorporating improved sequence features, and, critically, generating experimentally verified fungal EV datasets for model retraining and validation. Ultimately, the integration of computational prediction with targeted experimental confirmations may support the systematic characterization of EV proteomes in fungi.

## Figures and Tables

**Table 1:** Functional associations of CAZy families in the classical secretome of *M. circinelloides*.

Associated Function	CAZy Families	Number of Proteins
Fungal cell wall remodeling	CE4, GH18, GH20, GH72	40
Plant polysaccharide degradation	CE1, CE16, GH3, GH5, GH9, GH16, GH152, GH17, GH28, GH45, GH46, GH81, GH134, PL8, PL14	36
Glycan biosynthesis/modification	GH15, GH29, GH31, GH37, GH47, GH63, GT1, GT4, GT15, GT21, GT77	31
Carbohydrate-binding module	CBM1, CBM5, CBM18, CBM19, CBM20, CBM21, CBM48, CBM50	17
Auxiliary oxidative activities	AA1, AA2, AA3, AA5, AA12	9

## Data Availability

The original contributions presented in this study are included in the article/Supplementary Materials. Further inquiries can be directed to the corresponding authors.

## Supplementary Materials

The following supporting information can be downloaded at: https://www.mdpi.com/article/10.3390/jof12060442/s1, Figure S1: Structural prediction of representative CAZymes from *Mucor circinelloides*; Figure S2: Structural prediction of intrinsically disordered proteins from *Mucor circinelloides*; Table S1: SecSPI_predicted; Table S2: CAZymes_predicted; Table S3: nonclassical_predicted; Table S4: EV_predicted; Table S5: high_confidence_human_EV_dataset; Table S6: orthogroups_EV_validation.

## Abbreviations

The following abbreviations are used in this manuscript:

AA	Auxiliary Activity
AUC	Area Under the Curve
CAZyme	Carbohydrate-Active Enzyme
CBM	Carbohydrate-Binding Module
CE	Carbohydrate Esterase
ER	Endoplasmic Reticulum
EV	Extracellular Vesicle
GH	Glycoside Hydrolase
GT	Glycosyl Transferase
HMM	Hidden Markov Model
ML	Machine Learning
NN	Neural Network
PAE	Predicted Aligned Error
PL	Polysaccharide Lyase
pLDDT	Predicted Local Distance Difference Test
PTM	Post-Translational Modification
ROC	Receiver Operating Characteristic
TMH	Transmembrane Helix

## Appendix A

**Table A1:** List of extracellular Sec/SPI-negative proteins predicted to be secreted via non-classical pathways in *M. circinelloides*, including their SecretomeP NN scores, InterPro annotation status, and functional annotation.

Protein_ID	SecretomeP NN Score	InterPro Status	Annotation
EPB92339	0.919	No hit	-
EPB89683	0.88	No hit	-
EPB83760	0.826	No hit	-
EPB90535	0.824	No hit	-
EPB87768	0.811	Annotated	Phosphoesterase; Alkaline-phosphatase-like
EPB85967	0.803	No hit	-
EPB92617	0.797	No hit	-
EPB82815	0.774	No hit	-
EPB84464	0.717	No hit	-
EPB87726	0.709	Annotated	Peptidase S10, serine carboxypeptidase
EPB81863	0.695	Feature	consensus disorder prediction
EPB92248	0.682	Feature	consensus disorder prediction
EPB87963	0.64	Feature	consensus disorder prediction
EPB83393	0.611	Annotated	Peptidase S9, prolyl oligopeptidase
EPB85874	0.608	Annotated	Peptidase M13; Metallopeptidase
EPB81397	0.577	Annotated	Glycosyl hydrolase family 18; Chitinase
EPB82347	0.573	No hit	consensus disorder prediction
EPB92941	0.53	No hit	-
EPB81820	0.517	Annotated	MD-2-related lipid-recognition domain
EPB91055	0.509	Annotated	Aspartic peptidase A1
EPB83486	-	Annotated	Glycosyl hydrolase family 16; Beta-glucanase
EPB90259	-	Annotated	Glycoside hydrolase family 46; Chitosanase
EPB81606	-	Annotated	Armadillo-type fold
EPB81476	-	Annotated	Secreted LysM effector LysM1-like
EPB90311	-	Annotated	ATG15 Lipase; Fungal lipase-type domain
EPB87092	-	Feature	consensus disorder prediction
EPB82275	-	Feature	consensus disorder prediction
EPB83180	-	No hit	-
EPB81715	-	No hit	-
EPB82158	-	No hit	-
