# Supplementary material for: Machine-Learning Prediction of Extracellular Vesicle Protein Sorting Expands the Characterization of Secretory Functions in Mucor circinelloides
Source: J Fungi (Basel). 2026 Jun 17;12(6):442. doi: 10.3390/jof12060442 (PMC13301921; doi:10.3390/jof12060442)
Supplement: Supplementary file 1 [file jof-12-00442-s001.zip › Supplementary Figures.pdf]

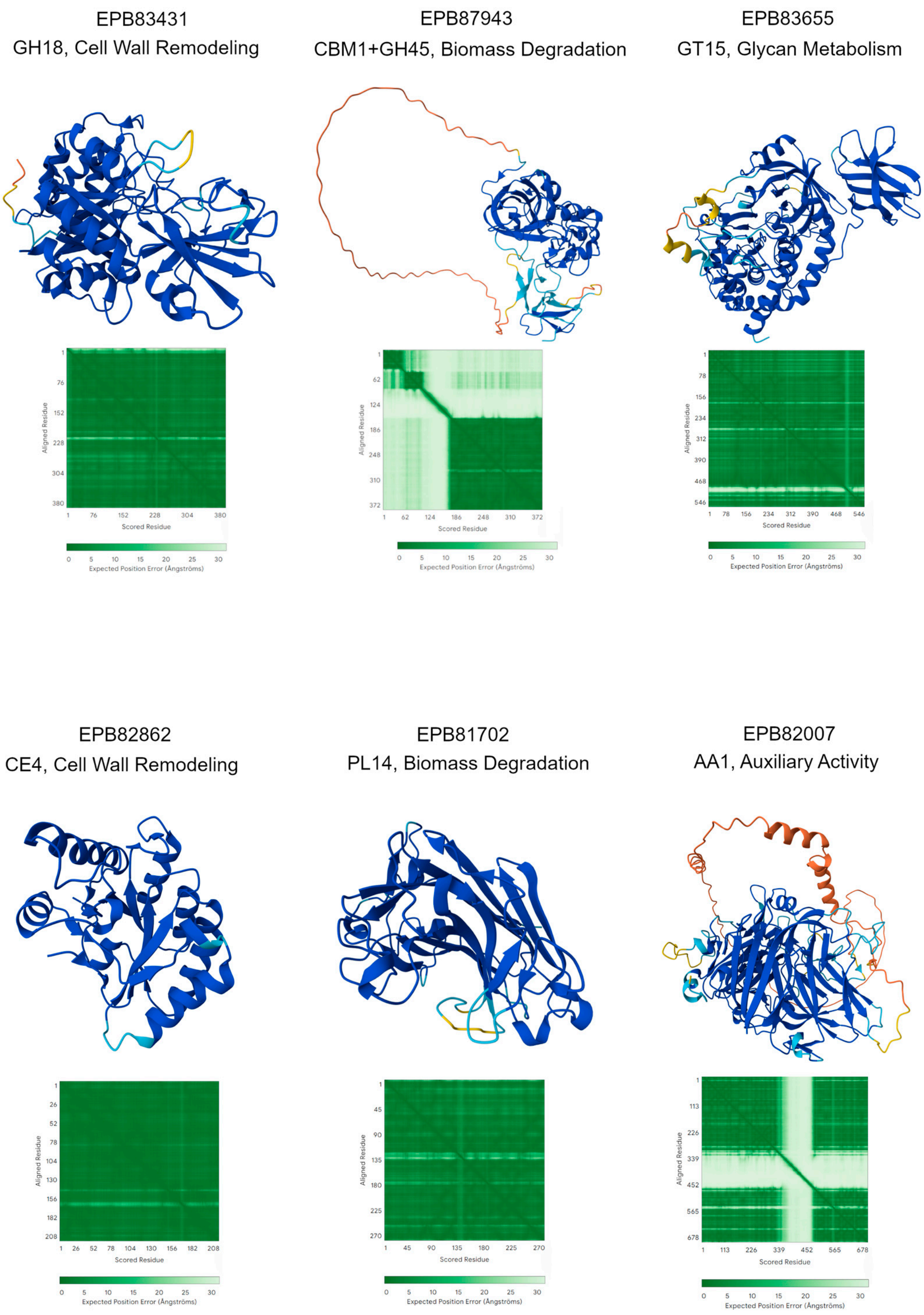

Figure S1. Structural prediction of representative CAZymes from *Mucor circinelloides*. AlphaFold-generated structural models of CAZymes selected based on their functional importance and representativity of the six major CAZy classes (GH, GT, CE, PL, AA, and CBM). The predicted structures exhibit high confidence, as indicated by overall well-defined folding patterns and low Predicted Aligned Error (PAE) graphs. Structural confidence is color-coded by pLDDT scores (dark blue, very high; light blue, high; yellow, low; orange, very low).

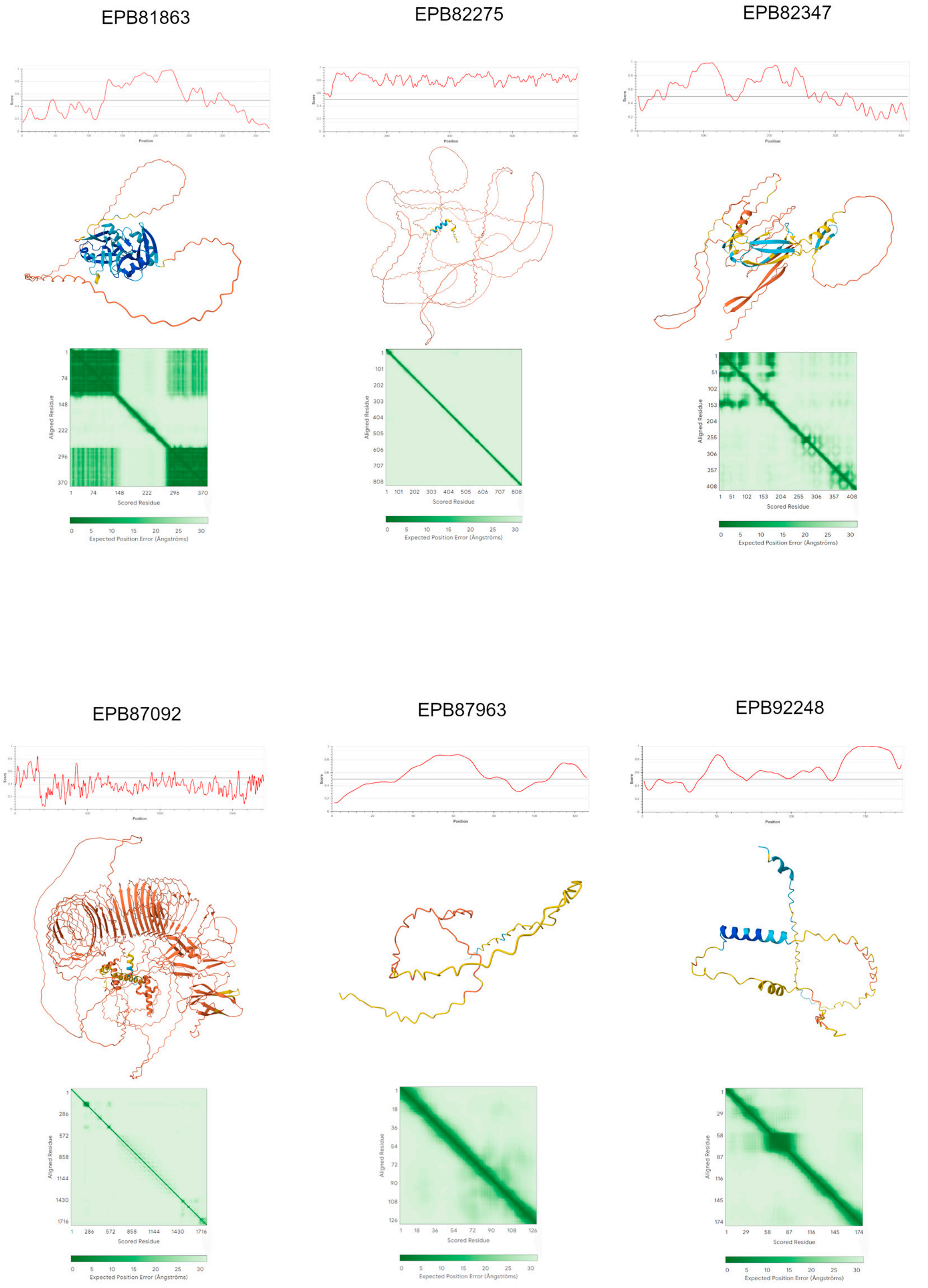

Figure S2. Structural prediction of intrinsically disordered proteins from *Mucor circinelloides*. AlphaFold-generated structural models of six proteins predicted to contain intrinsically disordered regions, supported by IUPred3 analysis. The predicted structures exhibit overall low confidence, with large portions displaying poorly defined folding patterns, as indicated by low pLDDT scores and high Predicted Aligned Error (PAE) graphs. Structural confidence is color-coded by pLDDT scores (dark blue, very high; light blue, high; yellow, low; orange, very low). Notable features include a partially structured region in EPB81863, a complex but low-confidence model in EPB87092, a small, structured segment in EPB82347, and predominantly disordered conformations in EPB82275, EPB7963, and EPB92248.
